# Measurement and Evaluation of the Gas Density and Viscosity of Pure Gases and Mixtures Using a Micro-Cantilever Beam

**DOI:** 10.3390/s150924318

**Published:** 2015-09-22

**Authors:** Anastasios Badarlis, Axel Pfau, Anestis Kalfas

**Affiliations:** 1 Laboratory of Fluid Mechanics and Turbomachinery, Aristotle University of Thessaloniki, GR-54124 Thessaloniki, Greece; E-Mail: akalfas@auth.gr; 2 R&D, Endress+Hauser Flowtec AG, Kägenstr. 7, CH-4153 Reinach, Switzerland; E-Mail: axel.pfau@flowtec.endress.com

**Keywords:** gas density, gas viscosity, AFM, micro-cantilever beam, piezo-resistive sensing

## Abstract

Measurement of gas density and viscosity was conducted using a micro-cantilever beam. In parallel, the validity of the proposed modeling approach was evaluated. This study also aimed to widen the database of the gases on which the model development of the micro-cantilever beams is based. The density and viscosity of gases are orders of magnitude lower than liquids. For this reason, the use of a very sensitive sensor is essential. In this study, a micro-cantilever beam from the field of atomic force microscopy was used. Although the current cantilever was designed to work with thermal activation, in the current investigation, it was activated with an electromagnetic force. The deflection of the cantilever beam was detected by an integrated piezo-resistive sensor. Six pure gases and sixteen mixtures of them in ambient conditions were investigated. The outcome of the investigation showed that the current cantilever beam had a sensitivity of 240 Hz/(kg/m${}^{3})$, while the accuracy of the determined gas density and viscosity in ambient conditions reached ±1.5% and ±2.0%, respectively.

## 1. Introduction

The information about density (ρ) and dynamic viscosity (μ) for the fluid of a process is important. This information can be used in the characterization, diagnostics and correction of the process. For example, by measuring the density of a gas for known pressure and temperature conditions, the composition of a binary mixture can be estimated [1]. The application area of these sensors can vary from the quality control of combustible gases to the characterization of DNA [2].

Micro-sensors can be defined as sensors that are orders of magnitude smaller in size than traditional sensors and, in many cases, are based on micro-system technology (MEMS). Micro-sensors for the measurement of density and viscosity present significant advantages, especially in gas applications, in comparison to the traditional methods, such as capillary sensors and vibrating wire [3], amongst others. Micro-sensors present a faster response time, easier integration, very high sensitivity and require a lower quantity of sample.

Over the last 20 years, a wide variety of geometry, material, sensing and excitation principles has been presented from different researchers in gas and liquid applications. Some sensors include micro-cantilevers for liquids [2,4,5,6] and for gases [7,8,9,10], quartz tuning forks for gas applications [1,11], membranes for liquids [12], plates [13] and quartz acoustic sensors [14]. The use of density and viscosity sensors for bio-applications is an emerging field of life science and includes sensors based on vibrating elements, which can be used for blood investigation [13] and other diagnostic purposes [15]. A detailed review of cantilever beams in bio-applications was published by Johnson *et al.* [16].

A gas viscosity density sensor was presented by Sell *et al.* [11], and the accuracy of this sensor was 0.2% in density and 2% in viscosity for densities higher than 3 kg/m${}^{3}$. This sensor was a quartz tuning fork, and it was investigated experimentally in three pure gases $N_{2},C_{2}H_{2},C_{3}H_{6}$. Goodwin [8] investigated a micro-plate in $Ar,N_{2},CH_{4}$. He showed that the accuracy of the sensor in the range of pressure between 1 and 8 MPa was 0.5% for density and 1.0% for viscosity.

Micro-cantilever beams have the advantage of a simple geometry that makes their fabrication significantly easier. Two very common excitation principles for a cantilever beam are the electromagnetic [8] and the piezoelectric principle. In the latter case, the piezoelectric element is integrated on the cantilever [17] or attached onto the base of a cantilever holder [5]. Another principle of excitation is the thermal method, which is based on the bimetallic effect. The heat can be induced by a laser [4] or by an integrated heater on its surface [18]. Finally, cantilever beams can also operate under electrostatic excitation [19].

Similarly, different deflection sensing approaches have been proposed. The most precise and accurate motion sensing approach is the optical principle [4]. On the other hand, piezo-resistors [20] have the advantage of easy integrability on the cantilever. Furthermore, piezo-electric [21] cantilever beams can use their activation element also for sensing by measuring conductance. The electrostatic principle presents exactly the same advantage, but its disadvantage is the small distance to the second electrode, which is usually attached to a wall. This generates an additional damping effect (squeeze film damping). Other researchers have also used electromagnetic read out [2] by measuring the induced alternating voltage on an integrated conductor of the cantilever. The substantial advantage of the electromagnetic read out is its simplicity, but its disadvantage is the low signal-to-noise ratio.

### 1.1. Current State of the Art

The flexural out-of-plane response of a cantilever beam can be modeled using two main approaches. The first is based on the simple harmonic oscillator theory (SHO), where the dynamic motion of the cantilever is modeled by the spring-dissipation-mass system approach and the response is described by an ordinary differential equation. The second approach uses the governing partial differential equation for the dynamic deflection of the beam that is based on the Euler–Bernoulli beam equation. The main dissipating mechanisms of a cantilever beam can be classified into three categories: viscous damping, thermoelastic damping and support losses.

The viscous effect in gaseous environments is the dominant factor of dissipation for micro-cantilever beams [22]. In the analysis where the SHO approach is adopted, the viscous effect is encapsulated in the overall quality factor. The first closed form solution for a vibrating sphere in viscous environments was developed by Landau *et al.* [6]. Blom *et al.* [22] used Landau’s model for the investigation of a silicon micro-resonator. Hosaka *et al.* [23] extended the model, representing the cantilever beam as a string of spheres and used Landau’s approach for the modeling of the viscous effects. Kirstein *et al.* [24] and Sader [25] modeled the cantilever using a vibrating cylinder in their analysis. The viscous effects formula was derived solving the linearized Navier–Stokes equations. Sader’s model for the frequency response of a cantilever beam was extended by Van Eysden *et al.*, firstly taking into consideration the torsion mode [26], secondly including in their model higher oscillating modes [27] and, finally, modeling the compressibility effects [28].

Thermoelastic damping and support losses become more important below ambient pressure. A model for support losses was proposed by Hao *et al.* [29], where they showed that the quality factor of support losses is proportional to the thickness-to-length ratio cubed. On the other hand, for the thermoelastic damping, an approximated thermoelastic damping model was presented firstly by Zener [30], while an exact solution was developed by Lifshitz *et al.* [31].

The scope of this study was to broaden the database on which model development is based and is tested. In addition, the validity of one of the currently-used models for the experimental quantification of density and viscosity was verified. Finally, this work intended to integrate the acquired knowledge of both experimental data, as well as modeling effort into a versatile instrument.

### 1.2. Current Approach

**Figure 1:**
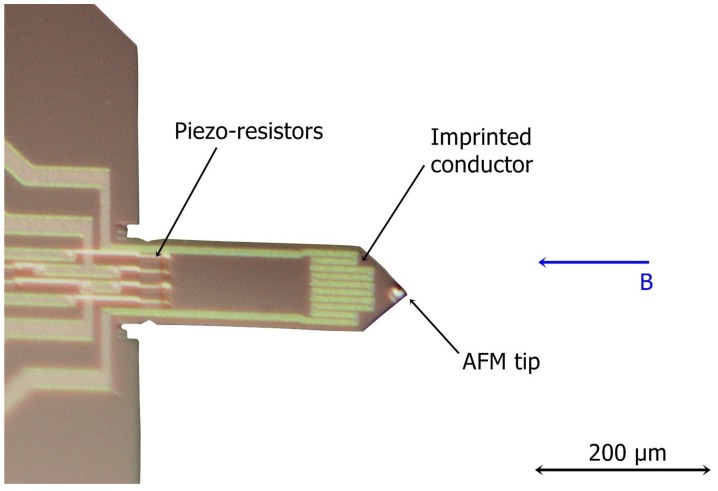
Micro-cantilever beam. At the free end, the AFM tip is barely distinguishable, while the imprinted aluminum conductor can be seen clearly. The imprinted conductor has a meandering form, due to the fact that the sensor was primarily designed to work with thermal excitation.

An atomic force microscopy micro-cantilever beam (Figure 1) was used in this investigation. The cantilever has an imprinted conductor and an integrated piezo-resistive bridge. The out-of-plane excitation was achieved using a permanent magnet and an AC current, which was applied to the imprinted conductor of the cantilever. This generated an electro-magnetic force, making the cantilever vibrate out of plane. The modeling approach was based on the simple-harmonic-oscillator (SHO), as this approach is applicable in real-time measurement. Firstly, the sensor was calibrated (adjusted) with four reference gases of known properties. Then, the sensor response was characterized for linearity and repeatability. Thirdly, the response of the sensor at higher modes was investigated. Finally, the sensor performance was experimentally verified by measuring six pure gases and sixteen mixtures.

## 2. Methods and Materials

### 2.1. Sensor

The sensor was an atomic force microscopy (AFM) cantilever beam, which was fabricated by SCL-Senso.Tech. Fabrication GmbH. The current sensor had a length of 310 μm, a width of 110 μm and a thickness of 3–5 μm, while the structural material of the sensor was Si. The cantilever beam has an aluminum imprinted conductor of 22 Ω, which primarily was designed to work as a heater. The sensing part of the sensor was a piezo-resistive bridge with four resistors. Each of the piezo-resistors had a resistance of 1 kΩ. From these four piezo-resistors, two were active and placed on the clamped side of the cantilever. A thorough characterization of a similar sensor was presented by Fantner *et al.* [18] and Burns [32]. The excitation principle in the current investigation was electromagnetic, placing the cantilever with its imprinted conductor in a constant magnetic field ($\vec{B}$), whose direction was parallel to the longitudinal axis of the cantilever and vertical to a part of the conductor on the free end. The excitation current had an amplitude of 1.25 mA, which guaranteed that there was no crosstalk between the excitation conductor and the piezo-resistive bridge [18]. The effect of the crosstalk between the excitation and the piezo-resistive bridge for this sensor was presented by Fantner *et al.* [18]. The crosstalk could be inductive, capacitive and thermal.

### 2.2. Experimental Setup

Six pure gases and sixteen of their binary mixtures were tested. The six gases under investigation were $He,Ar,N_{2},O_{2},CO_{2}$ and Ne. These six gases were selected due to the large range of property variation that they present, while they are not corrosive or explosive. The temperature ($T_{gas}$), pressure ($P_{gas}$) and the nominal density ($\rho_{gas}$) and dynamic viscosity ($\mu_{gas}$) of the gases can be found in Table 1. The nominal values for mixtures and pure gases were calculated using the PPDS software [33], which is a database for fluid properties developed by TÜV SÜD NEL. The investigated gases had no or a very low percentage of humidity. The maximum relative humidity that was recorded by the humidity sensor during the experiments did not exceed 2% in the worst case, while usually, it was below 0.5%. For this reason, in the above investigation, all of the gases were considered dry.

**Table 1:** Nominal gas properties.

*Gas*	$P_{\mathrm{gas}}\;$(bar)	$T_{\mathrm{gas}}\;$ (K)	$\rho_{\mathrm{gas}}\;$ (kg/m${}^{3}$)	$\mu_{\mathrm{gas}}\;$ (μPa·s)
$CO_{2}$	0.912	297.6	1.629	14.91
$CO_{2}-Ne\;73-27$	0.910	297.8	1.384	17.88
$CO_{2}-Ne\;52-48$	0.912	298.0	1.200	20.80
$CO_{2}-Ne\;33-67$	0.912	298.0	1.033	24.06
Ne	0.940	297.7	0.766	31.69
He	0.910	297.5	0.147	19.86
$N_{2}$	0.915	296.9	1.039	17.82
$He-CO_{2}\;50-50$	0.913	297.7	0.886	17.83
Ar	0.914	297.9	1.475	22.75
He-Ar32-68	0.912	298.2	1.047	23.47
$He-CO_{2}40-60$	0.913	297.8	1.033	17.14
He-Ar68-32	0.910	298.1	0.569	23.60
He-Ar11-89	0.954	296.8	1.392	22.94
$He-CO_{2}\;42-58$	0.912	297.8	1.003	17.28
He-Ar84-16	0.910	297.7	0.359	22.70
$O_{2}$	0.916	297.6	1.186	20.51
$CO_{2}-Ne\;16-84$	0.953	297.7	0.923	27.62
$CO_{2}-Ne\;84-16$	0.913	297.7	1.486	16.58
He-Ar50-50	0.911	298.2	0.807	23.72
$He-CO_{2}\;21-79$	0.943	298.0	1.357	15.99
He-Ar16-84	0.913	297.9	1.260	23.12
$N_{2}-O_{2}\;80-20$	0.914	297.3	1.065	18.36

**Figure 2:**
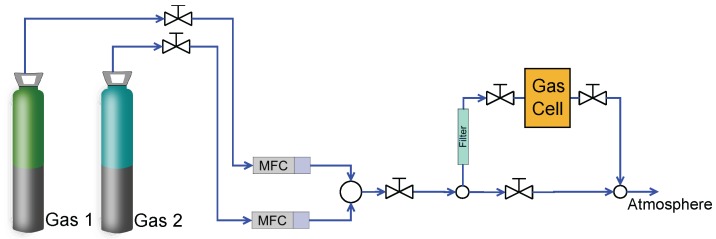
Schematic diagram of the experimental facility. The composition selection was carried out by controlling the flow through the mass flow controller; then, the two gases were mixed, and finally, the mixture was guided into the gas cell. During the measurement, there were no flow conditions in the gas cell.

In Figure 2, a schematic diagram of the experimental facility is presented. The composition selection was carried out by controlling the flow from the two gas bottles using two mass flow controllers (MFC). The accuracy of the mass flow controllers was 1%, as described by the manufacturer (Bronkhorst F-201CV). This inaccuracy in flow measurement generated an uncertainty in the composition of the mixtures. As illustrated, after the mass flow controller comes the mixing of the desired amount from each gas, and finally, the desired mixture fills the gas cell, where all of the sensors were placed. During the data acquisition process, the two valves of the gas cell (input and output) were closed, which guaranteed that the conditions of the experiments were steady and that no additional uncertainty was introduced into the system.

**Figure 3:**
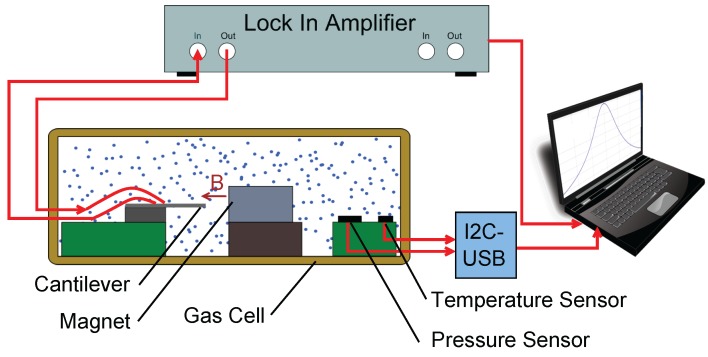
Schematic diagram of the experimental setup.

As is presented in Figure 3, the cantilever excitation (imprinted conductor) connectors were connected to the output of the lock-in-amplifier (HF2LI, Zurich Instrument) and the piezo-resistive bridge to the input. Specifically, the output of the bridge was connected to the differential input of the lock-in-amplifier. The input parallel resistor of the lock-in-amplifier had a value of 1 MΩ, while the output of the lock-in-amplifier had a resistance in series of 50 Ω. The lock-in-amplifier had sample rate of 210 millions of samples per second and an input resolution of 14-bit. During the measurement, the lock-in-amplifier recorded the amplitude and the phase shift of the piezo-resistive bridge. The communication protocol of the reference sensors was based on $I^{2}C$, and the values of the humidity, pressure and temperature were acquired with a $I^{2}C$ to USB interface. In the present investigation, the frequency response of the amplitude and the phase were recorded, alongside the temperature, pressure and humidity conditions.

**Figure 4:**
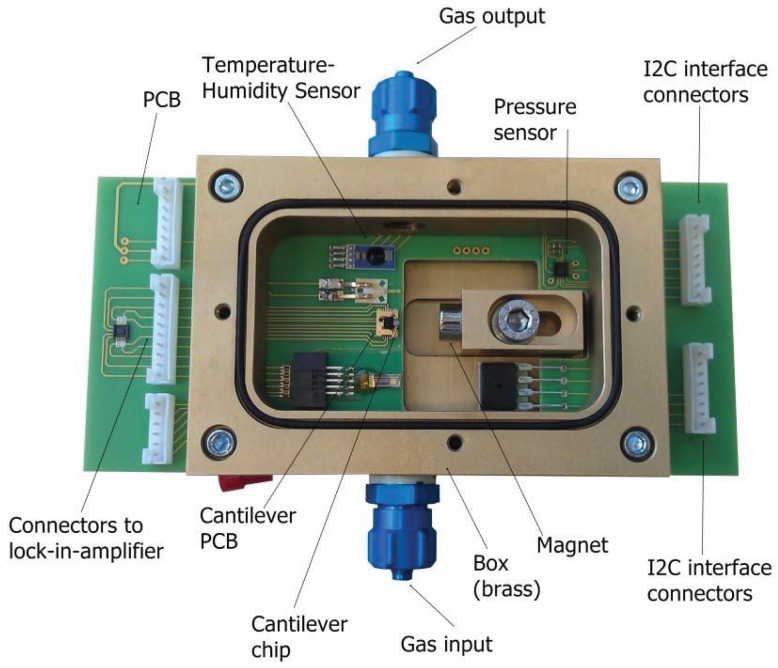
Gas cell photo. The micro-cantilever beam is mounted on the cantilever chip, which is mounted on the PCB. In addition, the magnet, the pressure, the temperature and the humidity sensors are displayed.

A photo of the gas cell can be seen in Figure 4. It consists of a brass box, a PCB, a magnet and the sensors. All of the sensors were mounted on a PCB. Brass was selected as the material for the box, due to its high thermal conductivity. The sensor under investigation was the micro-cantilever beam, which was built on the cantilever chip. In addition, temperature, humidity and pressure sensors were used as reference sensors. The magnet was placed on a slider, which made the distance from the cantilever adjustable.

A detailed schematic diagram of the gas cell and the electrical connections is presented in Figure 3. The cantilever was placed in a constant magnetic field, and the magnetic field vector was parallel to the longitudinal axis of the cantilever and vertical to the imprinted conductor at the free end, where the Lorentz force was generated by applying an alternating current of 1.25 mA through the conductor. The Lorentz force equation is presented in Equation (1),

(1) $$F_{L}=BIl_{conductor}=B\frac{V_{exc}}{R_{conductor}}l_{conductor}$$

where *B* is the magnetic flux density of the field, *I* is the current and $l_{conductor}$ is the length of the conductor, which was placed vertically to the magnetic field. This force made the cantilever vibrate in an out-of-plane (flexural) motion. The cantilever chip was soldered on a holder (cantilever PCB), and this holder was connected to the PCB. In the gas cell, there were also two reference sensors. These were a pressure meter (LPS331AP), which had a resolution and an accuracy of 0.020 mbar and 0.20 mbar, respectively, and a temperature-humidity sensor (HYGROCHIP HYT 271) with an accuracy of 0.2 K in temperature and 1.8% in the measurement of the relative humidity.

### 2.3. Modeling

**Figure 5:**
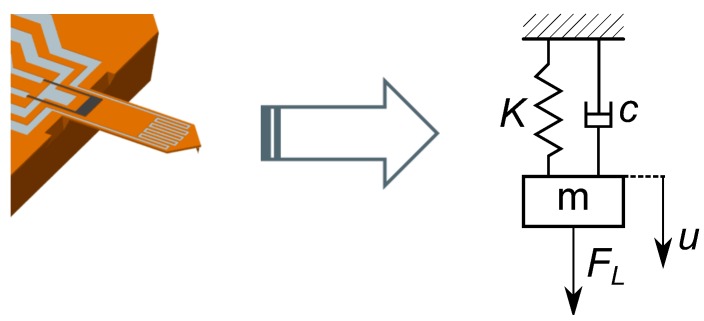
Cantilever beam and its simple harmonic oscillator representation, a system with mass, spring and damping. The external excitation is the Lorentz force on the imprinted conductor.

In the current investigation, the sensor was considered a simple harmonic oscillator (Figure 5). Using this approach, the equation of motion takes the form of Equation (2), where *u* represents the displacement of the mass from the balance position.

(2) $$m\frac{\partial^{2}u}{\partial t^{2}}+c\frac{\partial u}{\partial t}+Ku=F_{L}$$

The mass *m* of the oscillator represents the mass of the beam and the added mass of the gas (inertia effects). On the other hand, all of the damping mechanisms are encapsulated in the dissipation coefficient *c*. In addition, *K* is the spring constant of the beam, and $F_{L}$ is the electromagnetic external force that is applied to the vibrating body. In the current investigation, the excitation had a harmonic form with angular frequency *ω*, while its complex form was $F_{L}=F_{0}e^{j\omega t}$, where $F_{0}$ corresponds to the amplitude of the excitation force. Similarly to the excitation, the response of the cantilever was also harmonic. The harmonic response of the vibrating cantilever was derived by the exact solution of Equation (2) in the frequency domain. Rewriting Equation (2) in the frequency domain using the resonance frequency and the quality factor of the system, the equation takes the form:

(3) $$G\left(\omega \right)=\frac{\hat{F_{L}}\left(\omega \right)}{\hat{U}\left(\omega \right)}=K\left[1-\frac{\omega^{2}}{\omega_{0}^{2}}+j\frac{\omega }{Q\omega_{0}}\right]$$

In the latter equation, the angular resonance frequency is $\omega_{0}=\sqrt{K/m}$, the quality factor is $Q=m\omega_{0}/c$ and the spring constant is $K=bh^{3}E/\left(4L^{3}\right)$, where *h* is the thickness, *b* the width, *L* the length and *E* the elastic modulus of the cantilever beam.

The quality factor of the whole system was calculated adding the reverse individual quality factors for each dissipative mechanism of the system.

(4) $$\frac{1}{Q}=\frac{1}{Q_{Visc}}+\frac{1}{Q_{TED}}+\frac{1}{Q_{Support}}$$

As is presented in Equation (4), three main dissipation mechanisms contributed to the overall quality factor; the viscous effect due to the surrounding fluid, the thermo-elastic dissipation and the support losses.

The viscous effect is dependent on the properties of the surrounding fluid. The mechanism of viscous dissipation for the first out-of-plane flexural mode has been described by Sader [25]. The viscous quality factor can be calculated from equation:

(5) $$Q_{Visc}=\frac{\frac{4}{\pi \bar{T}}+\Gamma_{r}\left(Re_{S}\right)}{\Gamma_{i}\left(Re_{S}\right)}$$

where Γ(Re) is the hydrodynamic function for the rectangular beam, and the exact form can be found in Sader [25]. Equation (5) is dependent only on two natural dimensionless numbers, the Reynolds number $Re_{S}=\rho \omega b^{2}/\left(4\eta \right)$ and $\bar{T}=\rho b/\left(\rho_{b}h\right)$, where $\bar{T}$ expresses the ratio of added fluid mass to the mass of the beam. The three-dimensional flow effects at the tip of the cantilever were not taken into consideration, as there is no exact formula for the calculation of the *Q* at the tip.

Thermo-elastic dissipation is an intrinsic structural dissipation mechanism of oscillating elements. Mathematically, it can be expressed using the modeling approach from Zener [30]. The physical explanation of this dissipation mechanism is based on the coupling between the strain and the temperature field. Energy dissipation has the form of irreversible heat flow due to the local temperature gradient that accompanies the strain field through the coupling. The following equation calculates the thermo-elastic quality factor in a region around the first flexural mode for a isotropic homogeneous beam:

(6) $$Q_{TED}=\frac{\rho_{b}c_{p,b}}{E\beta_{b}^{2}T_{b}}\frac{1+{\left(\omega \frac{\rho_{b}c_{p,b}h^{2}}{\pi^{2}\lambda_{b}}\right)}^{2}}{\omega \frac{\rho_{b}c_{p,b}h^{2}}{\pi^{2}\lambda_{b}}}$$

where $\lambda_{b}$, $c_{p,b}$ and $\beta_{b}$ are the thermal conductivity, the specific heat capacity and the thermal expansion of the beam, while $T_{b}$ represents the temperature of the beam.

The third source of dissipation is the support losses on the fixed edge of the cantilever beam. Hao *et al.* [29] developed an analytical model for the calculation of the quality factor due to the support losses:

(7) $$Q_{Support}=k_{support}\frac{L^{3}}{h^{3}}$$

The value of the coefficient $k_{support}$ can vary, and researchers have proposed different values. In the current case, $k_{support}$ was considered equal to 0.34.

The interaction of the cantilever beam with the fluid leads to a frequency resonance shift. This resonance frequency shift can be calculated from Sader’s analysis [25], which states that the first flexural mode ratio of the resonance to the resonance frequency in a vacuum has the form of Equation (8):

(8) $$\frac{\omega_{0}}{\omega_{0,vac}}={\left(1+\frac{\pi }{4}\bar{T}\;\Gamma_{r}\left(Re_{S}\right)\right)}^{-0.5}$$

(9) $$\omega_{0,vac}=\frac{c_{n}^{2}}{L^{2}}h\sqrt{\frac{E}{12\rho_{b}}}$$

and is valid for homogeneous isotropic beams. In Equation (9), $c_{0}$ is 1.8751, which is the value for the first vibrating mode and is derived solving the eigen-problem of the Bernoulli–Euler equation.

Compressibility effects were considered negligible in this investigation, due to the fact that the sound wave length was much longer than the dominant length scale of the beam *b* [28]. The resonance of the first flexural mode was found in the frequency range of 34 kHz, and the expected sound wave length was 9714 μm, while the dominant characteristic length was 110 μm, which is two orders of magnitude lower than the sound wave length.

The above modeling approach is valid only for linear deflection of the cantilever. For this reason, the deflection of the beam free end did not exceed 2000 nm. In the following section, the linearity of the cantilever response is presented.

### 2.4. Quality Factor Measurement

The measurement of the quality factor was achieved by measuring the gradient of the phase at the resonance frequency position. The calculation formula is presented in Equation (10).

(10) $$Q_{meas}={\left|\frac{\partial \phi }{\partial f}\right|}_{f_{0}}\frac{f_{0,meas}}{2}$$

This method needs only two measurement points around the resonance frequency. Using this approach, the measurement process could be implemented using a phase-lock-loop (PLL) that follows the resonance frequency and measures the phase at one additional point very close to the resonance frequency. Then, the phase gradient can be calculated and, from the phase gradient, the quality factor (Equation (10)). Alternatively, if only amplitude data are available, a fitting of the amplitude data (Lorentzian fit) should be used for the extraction of the quality factor; then, during the measurement process, at least 30 points around the resonance frequency should be recorded and fitted.

**Figure 6:**
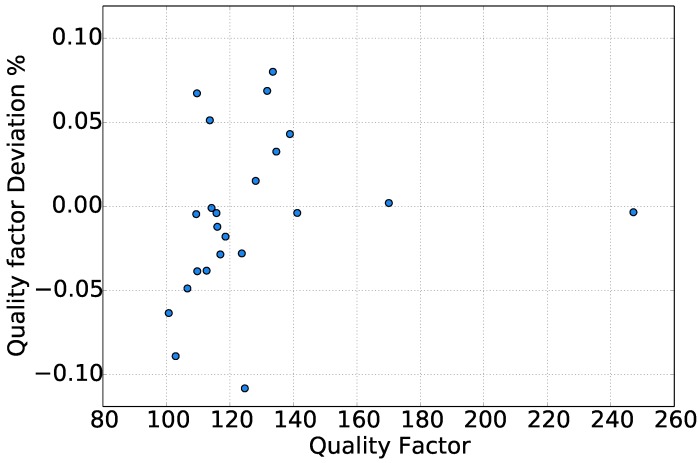
Quality factor deviation between the gradient method and the Lorentzian fit method.

A comparison of the measured quality factor is presented in Figure 6 between the gradient-based method and the Lorentzian fit method. The latter method is based on the fitting of the simple harmonic oscillator model to the spectrum of the amplitude of measured data, where finally, the quality factor was extracted. The deviation between the two methods did not exceed ±0.11%. For gases like helium and other gases with a high quality factor, the quality factor deviation was even lower than ±0.01%. The majority of the gases showed deviation below ±0.05%. The simple harmonic oscillator model manages to describe the response of the sensor accurately only in the range closest to the resonance frequency. For this reason, even in the Lorentzian fit of a SHO, if the range of fitting is extended far away from the resonance, the calculation of the quality factor is prone to error.

### 2.5. Calibration Measurement

**Figure 7:**
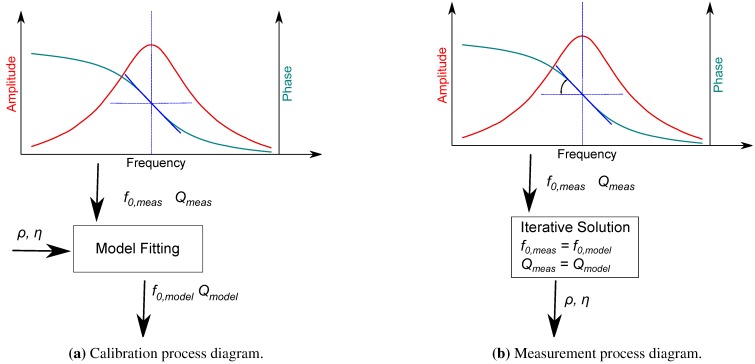
Calibration and measurement flowcharts.

As with every sensor, there was a discrepancy between the real behavior of the sensor and the model-predicted response. For this reason, calibration of the sensor is needed (Figure 7a). Calibration factors ($C_{1},C_{2},C_{3},C_{4},C_{5},C_{6}$) were added in Equation (4), Equation (5), Equation (6), Equation (7) and Equation (8), so these equations can be rewritten.

(11) $$\frac{\omega_{0}}{\omega_{0,vac}}=C_{1}{\left(1+\frac{\pi }{C_{6}}\bar{T}\;\Gamma_{r}\left(Re_{S}\right)\right)}^{-0.5}$$

(12) $$Re_{S}=\frac{\rho \omega b^{2}}{4\eta }C_{2}$$

(13) $$Q_{model}=Q\;C_{3}+C_{4}$$

(14) $$f_{0,model}=\frac{\omega_{0}}{2\pi }\left(\left(0.00055\left(\eta /1.9e^{-5}-1\right)+\frac{29.22}{500-Q_{model}}C_{5}\right)+1\right)$$

Equation (8) was replaced by Equation (11) adding one calibration factor as the multiplier ($C_{1}$) for the resonance angular frequency and replacing the constant value of the denominator with the calibration factor $C_{6}$. Calibration factor $C_{1}$ was used to adapt the gradient of the resonance angular frequency. The convention of the Reynolds number was also adapted, taking the form of Equation (12). The calibration factor $C_{2}$ on $Re_{S}$ was used for the adaptation of the hydrodynamic function. Two calibration factors $C_{3}$ and $C_{4}$ were introduced in order to adjust the quality factor in Equation (13), since a linear discrepancy was observed. Finally, the resonance frequency showed a systematic deviation in relation to the viscosity and the quality factor. In order to remove this systematic deviation, Equation (14) was added, containing also a calibration factor $C_{5}$.

The calibration factors were derived through an optimization process of the model with the data of the measurement (model fitting) using gases with known properties. A schematic diagram of the calibration process is displayed in Figure 7a. Through the optimization process, the measurement values of resonance frequency and quality factor were fitted to the model, respectively, for all of the calibration gases simultaneously. The model fitting process was a non-linear least squares problem, and the optimizer used was based on the Levenberg–Marquardt algorithm. In the current investigation four gas were used for the calibration of the sensor.

Similarly, no exact solution with respect to density and viscosity can be derived. This means that an iterative solution algorithm should be applied for the calculation of the gas density and viscosity in the measurement mode (Figure 7b). Initially, the resonance frequency and the quality factor of a gas were provided to the iterative solver. The solver used the Newton–Raphson scheme for the solution of the non-linear equations and the determination of the density and the dynamic viscosity.

## 3. Results Discussion

### 3.1. Sensor Characterization

**Figure 8:**
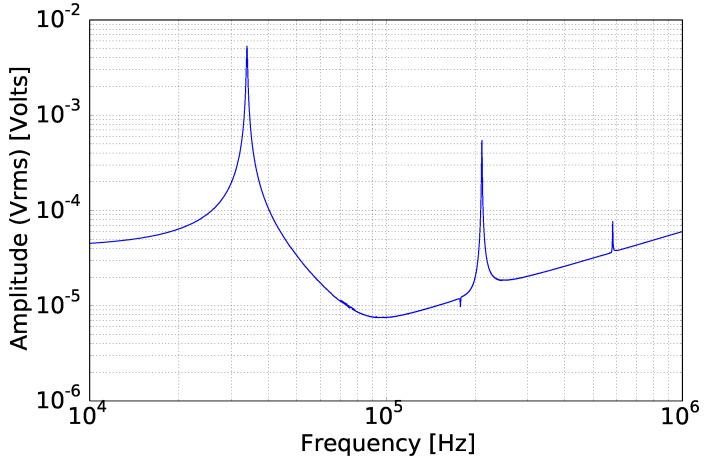
Amplitude of the micro-cantilever response in a spectrum. The first three flexural modes are clearly distinguishable.

**Figure 9:**
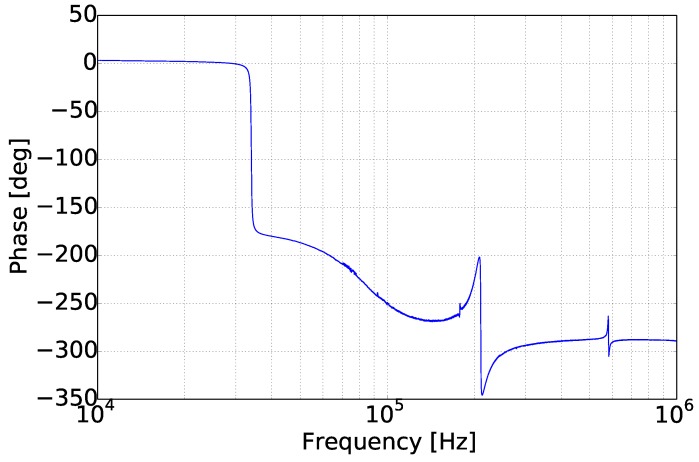
Phase shift of the micro-cantilever response.

By applying a frequency sweep in the range of 10 kHz–1 MHz in air, the response of the sensor was recorded by the lock-in-amplifier. Low frequencies were not measured, due to the high noise level and the low significance for the current investigation. In Figure 8 and Figure 9, where the amplitude and the phase of the cantilever are presented, three flexural mode peaks are distinguishable at 34 kHz, at 220 kHz and at 580 kHz. The recorded amplitude of the first mode is one order of magnitude higher than the second, while the third mode resonance peak is two orders of magnitude lower than the first. Another interesting observation, in Figure 8 and Figure 9, is a resonance peak in the negative direction in the range of 180 kHz, which also shows a phase shift of $15^{\circ }$. This effect is a torsional resonance mode, which slightly influences the piezo-resistors by reducing their imposed stress. From these three flexural modes, only the first has a phase shift of $180^{\circ }$, as was expected. In particular, the third flexural mode shows a very low phase shift of $40^{\circ }$.

Besides this effect, for frequencies higher than 100 kHz, the amplitude showed a linear relation to the frequency. This effect is not observed in the case of optical read out methods [18] and is related to the piezo-resistive read out method. The behavior of the cantilever beam response in the two latter observations was related to the inductive parasitic crosstalk effect between the piezo-resistor and the excitation conductor. The same behavior was observed by Naeli [20], where he had also used a piezo-resistive read-out. Riesch *et al.* [34] published a study, where this parasitic effect was approached by driving the piezo-resistive bridge with sinusoidal excitation. All of these effects introduced a difficulty in using piezo-resistive read-out for working modes higher than the third mode, as only the first mode was free of these effects, while the second and third are already influenced by these parasitic effects. In the current study, the cantilever was considered a simple harmonic oscillator, which is a one-degree-of-freedom system. In such a case, the higher-order flexural modes are neglected, as the first flexural mode is considered to dominate the response of the cantilever beam.

By comparing the ratio of the resonance frequencies for different modes between the experiments and the continuous system model (Euler–Bernoulli beam equation), Table 2 was derived. It is inferred that Equation (8) and its derived form:

(15) $$\frac{\omega_{n}}{\omega_{0}}={\left(\frac{c_{n}}{c_{1}}\right)}^{2}\frac{{\left(1+\frac{\pi }{4}\bar{T}\;\Gamma_{r}\left(Re,n\right)\right)}^{-0.5}}{{\left(1+\frac{\pi }{4}\bar{T}\;\Gamma_{r}\left(Re,1\right)\right)}^{-0.5}}$$

describe the cantilever behavior well. Equation (8) is derived from the continuous system of the fixed-free beam with fluid damping based on Sader’s model. As can be seen in Table 2, the measured ratio of the second to first resonance frequency deviates from the theory less than 1.5%, while that of the third to the first deviates less than 3.5%. This is in agreement with the observation that the Euler–Bernoulli beam equation deviates with increasing mode number. The first three values of the coefficients $c_{n}$ were calculated from the solution of the eigenvalue problem, which is derived from the Bernoulli–Euler equation. This coefficient takes the values of 1.875, 4.69 and 7.8611, respectively.

**Table 2:** Ratio of the second and third mode resonance to the first mode resonance.

	$\omega_{1,\mathrm{air}}/\omega_{0,\mathrm{air}}$	$\omega_{2,\mathrm{air}}/\omega_{0,\mathrm{air}}$
Theory	6.288	17.693
Measurement	6.204	17.138
Deviation	1.35%	3.23%

To avoid any geometric non-linearities, the deflection amplitude was kept low and did not exceed 2000 nm. A low deflection guaranteed that there was no geometric non-linearities on the response of the cantilever beam.

**Figure 10:**
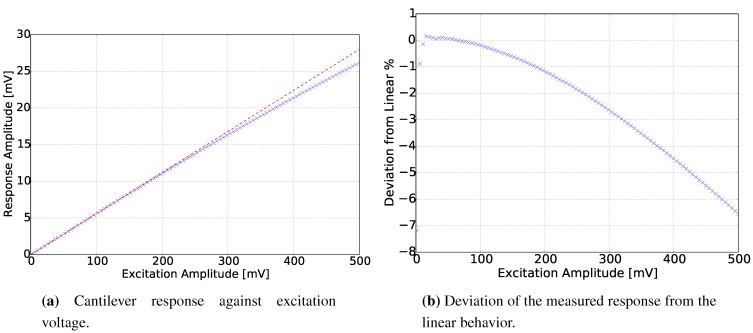
Sensor linearity.

Similarly, by sweeping the excitation voltage and recording the response amplitude of the cantilever beam, Figure 10a was derived. The process gas during this experiment was air, and the frequency of the excitation was 33.920 kHz, which is close to the resonance frequency of the cantilever beam in the case of air in ambient conditions. From Figure 10, it can be inferred that there are non-linear effects only in high excitation voltages. In Figure 10b, the deviation from linear stays below 1% for an excitation voltage up to 200 mV. For an excitation voltage above 100 mV, the deviation increases quadratically with the excitation voltage.

The causes of the non-linear behavior could be the inductive, the capacitive or the thermal crosstalk between the piezo-resistive bridge and the excitation conductor. Non-linear behavior could also have caused the deformation of the beam due to the bimetallic effect when the temperature of the cantilever increases. The excitation voltage had the harmonic form:

(16) $$V_{s}=V_{0}sin\left(\omega t\right)$$

where $V_{0}$ and ω are the excitation voltage amplitude and the angular excitation frequency, while $R_{lockIn}$ is the resistance in series of the lock-in-amplifier output. Although the cantilever was operating in low excitation voltages and using the electromagnetic excitation principle, the cantilever beam has an imprinted conductor heater. Consequently, as the excitation voltage increased, the heat generation on the conductor increased also quadratically.

(17) $$P=\frac{V_{0}^{2}R_{heater}}{2(R_{heater}+R_{lockIn})}-\frac{V_{0}^{2}R_{heater}}{2(R_{heater}+R_{lockIn})}cos\left(2\omega t\right)$$

Similarly, a quadratic deviation from the linear behavior is observed in Figure 10b. In addition to the thermal crosstalk, the thermal excitation has a steady and an alternating part (Equation (17)), which generates a steady and a periodic deflection, respectively. As is presented in Equation (17), this periodic deflection has a frequency that equals double the excitation frequency (second harmonic). From the above observations, it was concluded that the thermal effects had a dominant impact on the non-linearity of the cantilever response. To prevent all of the aforementioned effects, during the experiments with different gases, the excitation voltage remained low, with a value of 90 mV.

**Figure 11:**
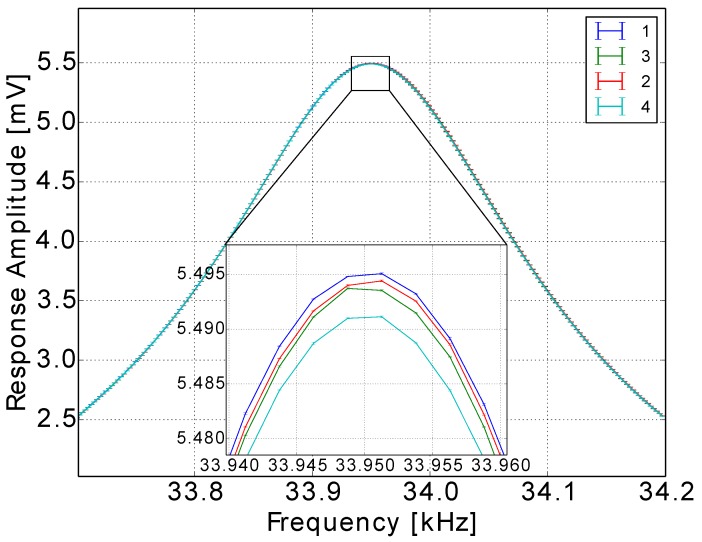
Repeatability investigations of the cantilever. The deviation between the curves was less than 0.07%, while the standard deviation (error bars) for each measurement point did not exceed 0.005%.

Besides the linearity verification, a repeatability check was conducted for the measurement electronics, the sensor and the whole system. The gas in the repeatability test was nitrogen; the gas pressure was 91,120 Pa; and the gas temperature was 298.15 K, while the percentage of humidity was below 2%. Running the same frequency spectrum four times (Figure 11) with the same gas and conditions, the curves showed only a 0.07% maximum deviation, which is the system uncertainty, while the standard deviation for each measurement point was less than 0.005%. The deviation of the system for the four measurements presented a systematic form. The very low repeatability error of the system and the sensor guaranteed a very low uncertainty in the measurement process from the side of the electronics and the sensor setup.

### 3.2. Response in Gases

**Figure 12:**
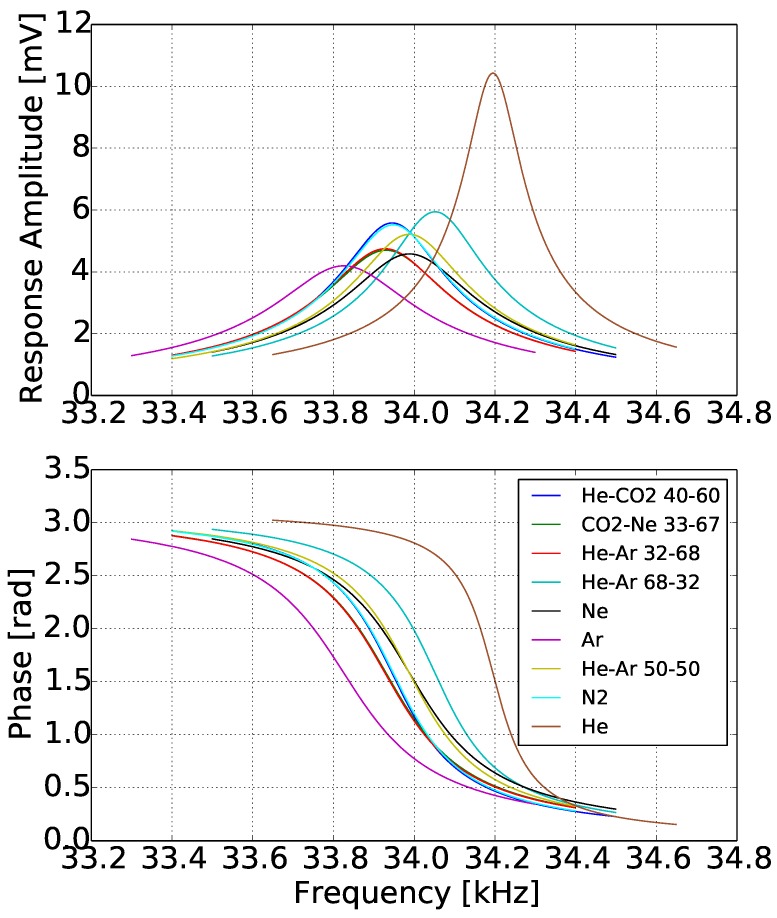
Frequency spectrum for different gases.

Figure 12 shows the response of the sensor in different gases, from a gas with very low density, in this case helium, to a gas with high density, like argon. The bandwidth of the cantilever frequency response was 1000 Hz for each gas around its resonance frequency. In this graph, the amplitude variation, the resonance frequency shift and the variation of the phase gradient at resonance frequency are distinguishable. The amplitude was high for the low density gases, for example helium had more than double the voltage amplitude of argon. The measured voltage amplitude was also symmetrically distributed around the resonance frequency, which was an indication that there were no parasitic effects. Another observation in Figure 12 can be seen in the phase graph, where as the density decreases, the phase shift around the resonance frequency takes place in a shorter bandwidth and is steeper. Similar observations can be made for the amplitude graph, where as the density decreases, the peak becomes sharper. The sharpness of the measurement peak is also reflected in the higher measured quality factor.

For a better understanding of the viscosity impact on the amplitude, a close look at the amplitude curves of He-Ar50-50 and Ne, shows that although the two gases have almost the same density, Ne has much higher viscosity, and this higher viscosity reduces significantly the amplitude resonance peak and makes it wider. From Figure 12, a general estimation of the sensor sensitivity with respect to density can be made. It is inferred that the sensitivity reached 240 Hz/(kg/m${}^{3})$. This very high sensitivity is due to the low mass and large width of the micro-cantilever beam.

**Figure 13:**
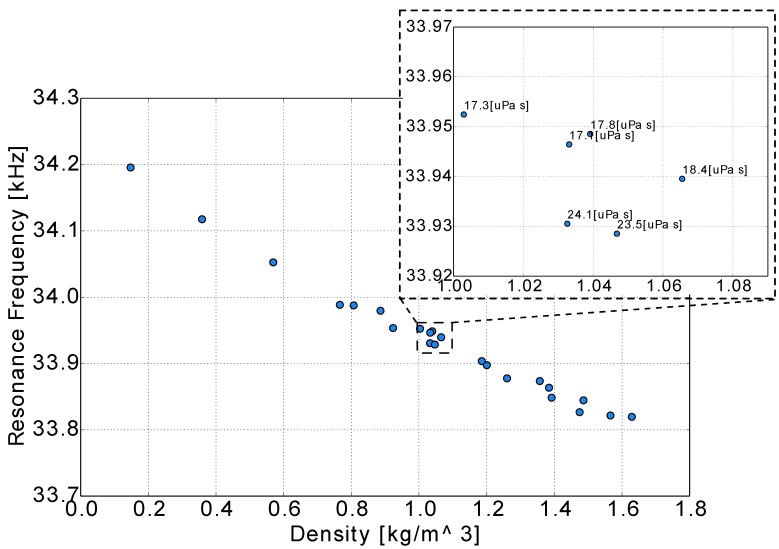
Resonance against density for different gases.

By plotting the resonance frequency for each gas against the density, Figure 13 was derived. In this figure, helium has the highest resonance frequency value, because it has the lowest density, whereas carbon dioxide has the lowest, which for this sensor and conditions has the value of 33,819 Hz. It is also inferred that there is an indication of a monotonic relation between the resonance frequency and the gas density.

For a better understanding of the viscosity impact on resonance frequency, four gases with a density of 1.04 kg/m${}^{3}$ (three mixtures and one pure gas) were measured. These four gases had the same density, but different viscosities. This difference led to a slight difference in resonance frequency, as presented in the magnified area of Figure 13. The gases with higher viscosity had a value of resonance frequency that was almost 17 Hz lower. Using a model that does not take the viscosity effect into consideration, it could lead to an error of 10% in the density measurement. Based on this observation, the need for a detailed model, which could handle all of the effects, was necessary for higher accuracy in density and viscosity measurement.

**Figure 14:**
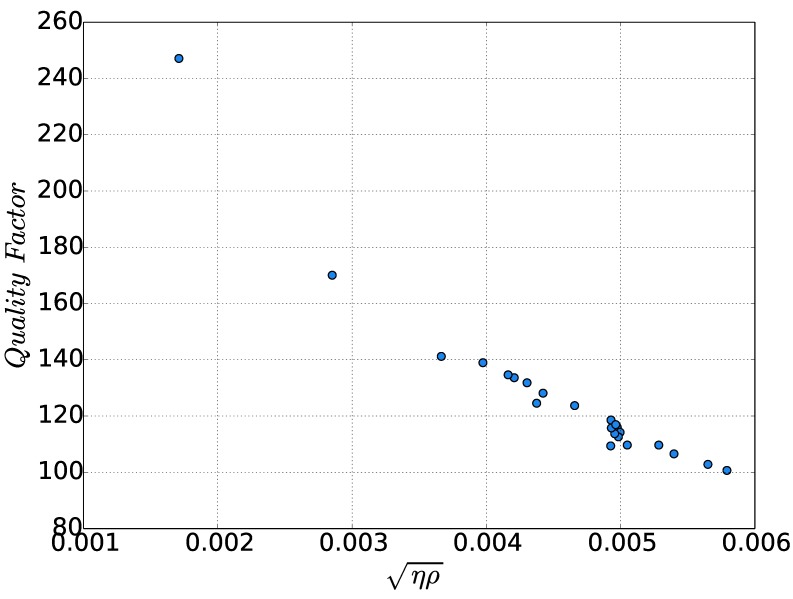
Quality factor against $\sqrt{\eta \rho }$ (kg s${}^{-0.5}$/m${}^{2}$).

On the other hand, the only quantity that follows a monotonic relation with the quality factor is the squared product of viscosity and density, as is displayed in Figure 14. From Figure 14, it is concluded that the quality factor was strongly dependent on both of the properties, while the sensitivity of the quality factor to the squared product of viscosity and density is higher for low values of this quantity.

### 3.3. Performance

For the verification of the model and the accuracy that this can deliver, a measurement campaign with 22 gases was conducted. The test gases were selected to cover a wide range of density and viscosity. The exact temperature and pressure for each gas measurement can be found in Table 1.

In Figure 7a, the calibration procedure of the sensor was presented. Following the sequence of Figure 7a and using four gases, the calibration coefficients were determined. The values for the specific sensor were $C_{1}:0.84473,C_{2}:0.4399173,C_{3}:1.1425,C_{4}:12.0811,,C_{5}:0.0096839,C_{6}:4.257249$. The calibration gases were He, He-Ar68-32, Ar and $CO_{2}$ and are marked with one additional cross symbol in the following figures.

**Figure 15:**
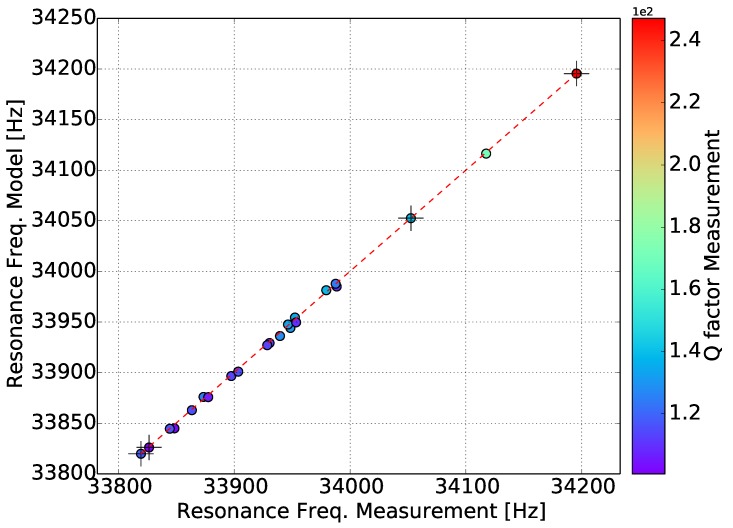
Resonance frequency experiments *versus* the model. The color of the points represents the measured quality factor.

**Figure 16:**
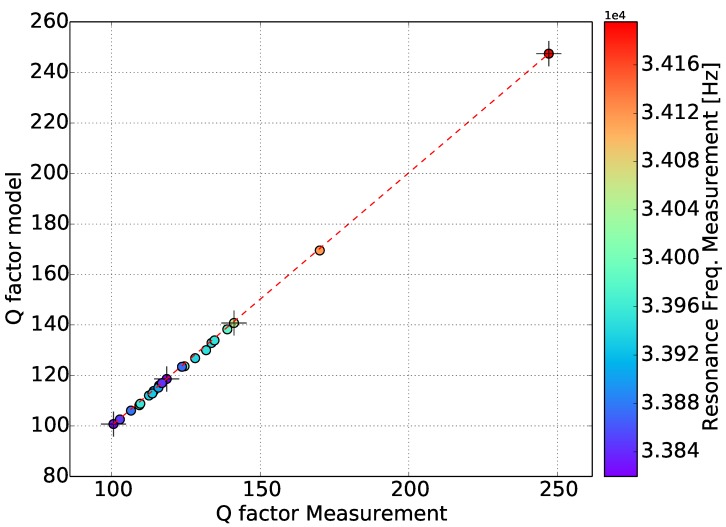
Quality factor experiments against the model. The color of the points represents the measured resonance frequency.

Figure 15 displays the measured against the model-determined resonance frequency. All of the gases fall into the same straight line, which is an indication that the calibrated model describes the real response of the sensor in gases with different densities and dynamic viscosity very well. Similarly, in Figure 16, the measured against the model-determined quality factor is plotted. Furthermore, in this case, all of the gases pass through the same straight line, which indicates that the model accurately represents reality. Helium has a higher quality factor value, while the majority of the gases are between 100 and 150.

**Figure 17:**
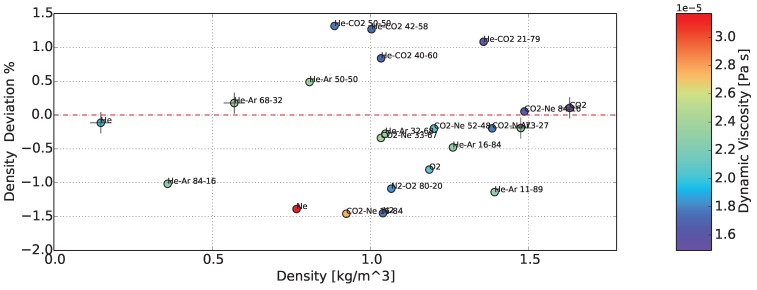
Deviation of the determined density from the nominal value. Calibration of the sensor in four gases (cross symbol). The color of the points represents the dynamic viscosity.

**Figure 18:**
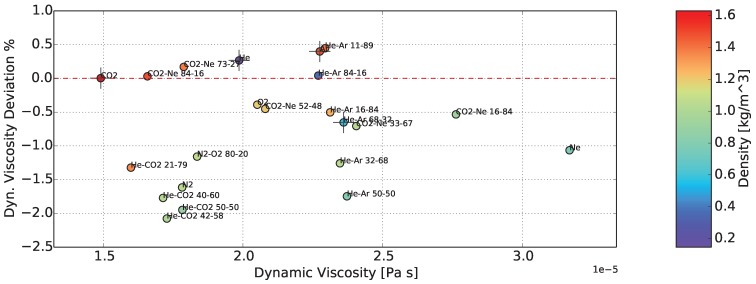
Deviation of the determined dynamic viscosity from the nominal value. Calibration of the sensor in four gases (cross symbol). The color of the points represents the density.

Finally, the accuracy or deviation from the nominal values of the sensor, in measuring the density and dynamic viscosity of gases, is demonstrated in Figure 17 and Figure 18. In the colored bars on the right-hand side, dynamic viscosity and density are displayed, respectively, as additional information. The achieved accuracy of density was better than ±1.5%, while for dynamic viscosity, it was between +0.5% and -2.0%. In dynamic viscosity, a systematic error was present, which has the form of an offset. The deviation from the nominal values is also presented in Table A1.

### 3.4. Uncertainty Analysis

The investigated gases can be classified into two categories; mixtures and pure gases. The dominant uncertainty factor of pure gases was the uncertainty in the measurement of the resonance frequency. This uncertainty was related to the used resolution of the frequency sweep. On the other hand, the reference values of density and viscosity for gas mixtures contained two additional sources of uncertainty; these are the accuracy of the mixture database and the real composition that the test gas had. Composition uncertainty was introduced due to the inaccuracy of the mass flow controllers, which were used to control the flow from each gas bottle. The other uncertainty factors, like the pressure uncertainty and the ambient temperature uncertainty for the reference values, had a significantly lower impact. The overall uncertainties, in the case of the mixture and pure gases, are also added in the last two columns of the Table 3. They were calculated from the partial uncertainties using the formula of error propagation for a function with several variables. The overall and the partial uncertainties can be seen in Table 3.

**Table 3:** Uncertainty of the sensor for the measurement of density and viscosity.

	$\delta T_{\mathrm{gas}}$	$\delta P_{\mathrm{gas}}$	$\delta f_{0}/f_{0}$	δϕ/ϕ	Mixture	Mixtures	Overall	Overall
	(0.2 K)	(20 Pa)	$\left(1.5e^{-5}\right)$	$\left(1.3e^{-4}\right)$	Database	Composition	Mixtures	Pure Gases
δρ/ρ%	–0.066	0.02	0.8	0.03	2	1	2.376	0.8035
δη/η%	0.052	$1.9e^{-7}$	0.2	0.03	2	1	2.246	0.2088

## 4. Conclusions

In the present investigation, the performance of an AFM cantilever beam in measuring gas density and viscosity for a range of 22 gases was presented. The objective of this work was to investigate the sensitivity and the accuracy that can be achieved using a micro-cantilever beam from AFM as a sensor in a wide range of gases. The modeling of the sensor was based on the simple harmonic oscillator approach. The micro-cantilever beam showed very high sensitivity, which was higher than 240 Hz·m${}^{3}$/kg. On the other hand, the accuracy of the sensor in density was better than ±1.5% and in dynamic viscosity was between +0.5% and -2.0%. Although the current investigation was conducted in ambient pressures, where the density of the gases is very low, the sensor succeeded in accurately measuring gases with very low density. Using the simple harmonic oscillator modeling approach, the measurement could be done in real time, as only the resonance and the quality factor are needed for the determination of the density and dynamic viscosity. Based on all of the above observations, the micro-cantilever beam structure is the ideal core element of a density and viscosity instrument, which is intended to operate in gas applications.

## Appendix

The resonance frequency, the quality factor and the deviations of density and viscosity from the nominal values are also provided in Table A1 to document the experimental data for the future reference of the interested reader.

**Table A1:** Resonance frequency, quality factor and achieved deviation of the density and dynamic viscosity for all of the gases under investigation.

*Gas*	$f_{0,\mathrm{meas}}\;$ (kHz)	$Q_{\mathrm{meas}}\;\left(\;\right)$	$\left(\rho -\rho_{\mathrm{nom}}\right)/\rho_{\mathrm{nom}}\;\%$	$\left(\mu -\mu_{\mathrm{nom}}\right)/\mu_{\mathrm{nom}}\;\%$
$CO_{2}$	33.820	118.59	0.11	0.00
$CO_{2}-Ne\;73-27$	33.863	116.02	−0.20	0.17
$CO_{2}-Ne\;52-48$	33.897	114.22	−0.20	−0.45
$CO_{2}-Ne\;33-67$	33.931	112.61	−0.34	−0.71
Ne	33.988	109.39	−1.39	−1.07
He	34.196	247.12	−0.12	0.27
$N_{2}$	33.949	131.83	−1.45	−1.62
$He-CO_{2}\;50-50$	33.979	138.97	1.32	−1.95
Ar	33.827	100.67	−0.19	0.40
He-Ar32-68	33.929	113.74	−0.28	−1.26
$He-CO_{2}\;40-60$	33.946	133.64	0.84	−1.77
He-Ar68-32	34.053	141.21	0.18	−0.65
He-Ar11-89	33.848	102.83	−1.14	0.45
$He-CO_{2}\;42-58$	33.952	134.66	1.27	−2.08
He-Ar84-16	34.118	170.07	−1.02	0.04
$O_{2}$	33.904	115.78	−0.81	−0.39
$CO_{2}-Ne\;16-84$	33.953	109.70	−1.46	−0.53
$CO_{2}-Ne\;84-16$	33.844	116.96	0.05	0.03
He-Ar50-50	33.988	124.57	0.49	−1.75
$He-CO_{2}\;21-79$	33.874	123.72	1.08	−1.32
He-Ar16-84	33.878	106.56	−0.48	−0.5
$N_{2}-O_{2}\;80-20$	33.940	128.14	−1.09	−1.16
